# Beef Nutritional Characteristics, Fat Profile and Blood Metabolic Markers from Purebred Wagyu, Crossbred Wagyu and Crossbred European Steers Raised on a Fattening Farm in Spain

**DOI:** 10.3390/ani13050864

**Published:** 2023-02-27

**Authors:** Juan M. Vázquez-Mosquera, Aitor Fernandez-Novo, Eduardo de Mercado, Marta Vázquez-Gómez, Juan C. Gardon, José L. Pesántez-Pacheco, Ángel Revilla-Ruiz, Raquel Patrón-Collantes, Maria L. Pérez-Solana, Arantxa Villagrá, Daniel Martínez, Francisco Sebastián, Sonia S. Pérez-Garnelo, Susana Astiz

**Affiliations:** 1 Medicine and Surgery Department, Veterinary Faculty, Complutense University of Madrid, Puerta de Hierro Avenue s/n, 28040 Madrid, Spain; 2 Department of Veterinary Medicine, School of Biomedical and Health Sciences, Universidad Europea de Madrid, C/Tajo s/n, 28670 Villaviciosa de Odón, Spain; 3 Animal Reproduction Department, National Institute of Agronomic Research (INIA-CSIC), Puerta de Hierro Avenue s/n, 28040 Madrid, Spain; 4 Nutrition and Obesities: Systemic Approaches Research Unit (NutriOmics), Institut national de la santé et de la recherche médicale (INSERM), Sorbonne Université, 75006 Paris, France; 5 Department of Animal Medicine and Surgery, Veterinary and Experimental Sciences School, Catholic University of Valencia-San Vicente Mártir, Guillem de Castro, 94, 46001 Valencia, Spain; 6 School of Veterinary Medicine and Zootechnics, Faculty of Agricultural Sciences, University of Cuenca, Doce de Octubre Avenue, Cuenca 010150, Ecuador; 7 Institut Valencià d’Investigacions Agràries (IVIA), CV-315, Km 10,7, 46113 Valencia, Spain; 8 Embriovet SL, Polígono Industrial de Piadela II-8, 15300 Betanzos, Spain; 9 Cowvet SL, País Valenciano Avenue 6, 46117 Betera-Valencia, Spain

**Keywords:** Black-Japanese, angus, oleins, MUFA, PUFA, amino acids, health-related indexes

## Abstract

**Simple Summary:**

Beef cattle production has improved to achieve consumers’ preferences, including meat quality and human-health-related indexes. Wagyu (WY) breed is Japanese cattle with high intramuscular fat infiltration and rich in unsaturated fatty acids. Most Wagyu beef cattle are raised in Japan. Our objective was to describe Wagyu, Wagyu-by-Angus (Wangus, WN), and Angus-by-Charolaise-Limousine (ACL) beef produced in a Spanish fattening system with high-olein diets, regarding the fat profile, health-related indexes and the metabolic biomarkers prior to slaughtering. Blood lipid-related metabolites, except for non-esterified fatty acids (NEFA) and low-density level cholesterol (LDL), were higher in WY and WN than in ACL, while glucose was lower in WY and WN. Leptin was higher in WN than in ACL. Nutritional analyses showed higher fat infiltration in WY and WN steers than ACL animals for both meat cuts (sirloin and entrecote), including three-fold higher content. Wagyu beef had the highest intramuscular fat in sirloin (51.5% vs. 21.9%) and entrecote (59.6% vs. 27.6%) vs. ACL animals. Wagyu entrecote contained more unsaturated fatty acids (55.8% vs. 53.0%) and more oleic acids (47.5% vs. 43.3%) than ACL’ beef. Wagyu and WN entrecote showed better atherogenic (0.6 and 0.55 vs. 0.69), thrombogenicity (0.82 and 0.92 vs. 1.1), and hypocholesterolemic/hypercholesterolemic index (1.9 and 2.1 vs. 1.7; all *p* < 0.001) than ACL’s beef. In brief, the fat profile and nutritional characteristics of beef depend on the fattening period, breed/crossbred, and cut of meat, with Wagyu and Wangus beef showing a healthier fat profile than ACL animals.

**Abstract:**

A high intramuscular fat content characterizes Wagyu (WY) cattle breed. Our objective was to compare beef from WY, WY-by-Angus, or Wangus (WN) steers with European, Angus-by-Charolais-Limousine crossbred steers (ACL), considering metabolic biomarkers pre-slaughtering and nutritional characteristics, including health-related indexes of the lipid fraction. The fattening system with olein-rich diets and no exercise restriction included 82 steers, 24 WY, 29 WN, and 29 ACL. The slaughter ages and weights were (median and interquartile range) 38.4 mo.-old (34.9–40.3 mo.) and 840 kg (785–895 kg) for WY; for WN, 30.6 mo. (26.9–36.5 mo.) and 832 kg (802–875 kg), and for ACL steers, 20.3 mo.-old (19.0–22.7 mo.) and 780 kg (715–852 kg). Blood lipid-related metabolites, except for non-esterified fatty acids (NEFA) and low-density level cholesterol (LDL), were higher in WY and WN than in ACL, while glucose was lower in WY and WN. Leptin was higher in WN than in ACL. Pre-slaughtering values of plasma HDL underscored as a possible metabolic biomarker directly related to beef quality. The amino-acid content in beef did not differ among experimental groups, except for more crude protein in ACL. Compared to ACL, WY steers showed higher intramuscular fat in sirloin (51.5 vs. 21.9%) and entrecote (59.6 vs. 27.6%), more unsaturated fatty acids in entrecote (55.8 vs. 53.0%), and more oleic acid in sirloin (46 vs. 41.3%) and entrecote (47.5 vs. 43.3%). Compared to ACL entrecote, WY and WN showed better atherogenic (0.6 and 0.55 vs. 0.69), thrombogenicity (0.82 and 0.92 vs. 1.1), and hypocholesterolemic/hypercholesterolemic index (1.9 and 2.1 vs. 1.7). Therefore, beef’s nutritional characteristics depend on breed/crossbred, slaughtering age and cut, with WY and WN entrecote samples showing a healthier lipid fraction.

## 1. Introduction

The Black-Japanese breed, also called Wagyu, is known worldwide because of the outstanding quality and organoleptic characteristics of its meat due to its extensive fat infiltration of muscle [1,2,3]. Gotoh et al. [4] determined a very high level of intramuscular fat content (IMF) in the *Longissimus thoracis et lumborum* muscle of 24 months old Wagyu steers (23%), while German Angus, Belgian Blue, and Holstein Friesian bulls showed lower IMF (<5%). Wagyu cattle have not only higher IMF [5] but also a different fatty acid composition (FA) with a larger oleic acid (OA) concentration (52.9% of total fatty acids) when compared to Hanwoo steers (47.3%) or corn-fed Angus (39.8%) [6]. Meat chemical composition and the FA profile are directly linked to beef quality [7], with an increased crude fat content (range 23.8–48.6%) improving tenderness, juiciness, and fattiness [5] and thereby its acceptability by the consumers in Japan [2].

Consumer concerns are also linked to issues related to the health and convenience of beef intake [8] and the advantages and disadvantages of red beef consumption [9]. Although beef is a nutrient-dense protein source, the saturated fat content has been associated with an increased risk of cardiovascular disease [10], diabetes type 2 [11], and mortality due to non-communicable diseases [12]. Contrary to these associations, clinical trials have demonstrated that the inclusion of beef in a healthy and balanced diet does not negatively influence disease risk factors [13]. Furthermore, a recent meta-analysis shows that out of the top 15 dietary components related to non-communicable disease risk, a diet high in red meat was ranked as the lowest dietary factor related to increased risk [14]. Due to the high OA content, Wagyu beef has even been related to a reduction in the blood cholesterol level in human consumers [15]. Therefore, besides the quantity, the lipid composition of meat is of increasing interest not only due to its effect on the organoleptic characteristics of the meat [16] but to rate how healthy beef is [17,18]. In fact, cardiovascular disease and coronary heart disease risk are reduced when saturated fatty acids (SFA) are replaced by monounsaturated fatty acids (MUFA) and polyunsaturated fatty acids (PUFA) [13]. These effects can be achieved by modifying the profile of the ingested fat [19] and the kind of beef consumed [20,21,22].

The effects of the diets may vary depending on the bovine breed [23]. Key fatty acid metabolism-associated genes with upstream regulators (SREBF1) are related to a higher FA index in beef [24]. In the Wagyu breed, condensed barley distillers soluble promoted growth, a higher glycolytic and lower oxidative muscle metabolism [25], with this breed steers producing higher MUFA percentage in carcasses than Angus steers when fed high-roughage diets [26]. Increasing the concentration of oleic acid in diet, Wagyu beef increased the OA level in IMF [6], but there is no evidence about Wagyu- and Angus-crossbreed managed under Spanish farming conditions. The evidence suggests that depending on the breed, the nutritional system can induce relevant improvement in the beef fatty acid profile.

Nutritional and management systems trigger changes in the homeostasis of the fattened animals, inducing blood metabolite changes [27]. These changes may be the link between management and final beef characteristics. Indeed, metabolite concentrations are commonly used to assess health and nutritional status in cattle [28,29]. In Wagyu cattle, metabolic markers show different patterns than in another leaner breeds [30,31]. The main metabolic parameters related to marbling are cholesterol, glucose, and urea, among others [31,32,33,34,35,36]. Although these parameters may change with fed diets, the relationship among metabolic parameters, beef composition, and fatty acid profile remains unclear.

A previous study of our group described healthy growing purebred Wagyu, crossbred Wagyu-by-Angus (Wangus), and Angus-by-European crossbreeds, such as Charolais and Limousine (ACL) steers, in a Spanish fattening system involving olein-rich diets, no exercise restriction and high animal welfare [37]. We hypothesize that this production system results in different beef products depending on the experimental group, in fact, on the breed/crossbred. Furthermore, the aim of the study was to describe the beef characteristics of these groups raised in this Spanish fattening system and to explore the relationship between blood metabolic parameters prior to slaughtering and beef characteristics, possibly associated with the group and age at slaughtering. This information is highly relevant to choose the optimal system to produce a more appreciated, high-marbled beef with Wagyu animals efficiently in other world regions outside Japan.

## 2. Materials and Methods

### 2.1. Animals and Farm Management

This study was conducted under commercial farming practices on the farm “Mudéjar-Wagyu”, located in the North-Centre of Spain, with the appropriate farming measures and clinical activities [38]. All available, healthy steers slaughtered during the study time were included in the study. A total of 82 steers were studied: 24 purebred Black-Japanese or Wagyu, (WY); 29 crossbred Wagyu-by-Angus or Wangus (WN); and 29 Angus-by-European Charolais and Limousine crossbred steers (ACL). Data were recovered at once, at the end of the fattening period, without repeated measurements. A different objective-slaughtering age was intended by experimental group, following the usual slaughtering times. This guaranteed an adequate carcass quality, with an earlier expected slaughtering age in European crossbreds, to avoid excessive fattening (WY ≈ 37 mo., WN ≈ 31 mo., and ACL ≈ 22 mo.-old), assuring a comparable and marketable beef after slaughtering the steers from the three experimental groups. The actual slaughter ages, expressed as median and interquartile range, were 38.4 mo.-old (34.9–40.3 mo.) for WY; for WN, 30.6 mo. (26.9–36.5 mo.) and for ACL steers 20.3 mo.-old (19.0–22.7 mo.).

The animals were raised as described previously [37]. Castrated male calves were housed during the last phase of fattening in groups of 10 animals/pen (20 m^2^/animal). All barns were open (natural ventilation), with anti-slip, concrete or soil floors and chopped straw bedding, and access to open areas. Brushers and sprinklers were available in the pens assuring a high level of animal welfare. The nutritional management of the animals included ad libitum water and diets adjusted to their requirements [39]. From 10 to 22 mo. of age, the diet was a wet total mixed ration (wet TMR), and afterward, the diet was a dry TMR enriched in oleins. ACL animals received the finalization diet at least 2 months before slaughtering, independently of age. Further details on diets and management are described in the previous study on productive and health parameters [37], and the detailed composition of this finalization diet, enriched in oleins, is resumed in Table 1 and Table 2.

Animals were slaughtered according to European Union regulation 1099/2009. Carcasses were aired for temperature decrease up to 4 °C in less than 24 h and kept at 2 °C for 72 h. At the meat processing facility, 200 g fresh meat samples of the Longissimus lumborum muscle, between the lumbar ribs (sirloin) and Longissimus thoracis between the 6 and 7th ribs (entrecote or ribeye steak) were taken from each carcass on the 5th day after slaughtering. Samples were adequately identified, vacuum-packed, and sent under a stable temperature of 4 °C to the diagnostic laboratory (Labocor Analítica, Colmenar Viejo, Madrid, Spain), operating under the Spanish National Accreditation Body (ENAC; complying the applicable requirements and conditions of the standard GMP+ B10 Laboratory Testing of the GMP+ FC scheme (based on GMP+ C6) of GMP+ International and the UNE-EN ISO/IEC 17025:2017).

### 2.2. Description of the Slaughtered Animals: Weight, Height and In Vivo Fatness

Animals that could enter the weighing booth during the last week before slaughtering (n = 15/24 WY, 14/29 WN, and 29/29 ACL) were weighted (BW, Kg) at the farm before slaughtering using an electronic balance (TRU–TEST, Auckland, New Zealand; accuracy of ±0.5 Kg), while its height (cm) was determined with the animal standing, using a measuring stick, from a point directly over the hip bones (hocks) to the floor. In vivo evaluation of the body-fattening stage was performed with ultrasound using the PieQuip technology and a MyLab One^®^ ultrasound system (Esaote, Barcelona, Spain) with an animal science probe (ASP) of 18 cm length and a frequency of 3.5 Mhz linear transducer [40,41]. Measurements were made at the closest moment before slaughtering when images could be processed. As previously determined [37], in Wagyu and Wangus animals older > 22 mo. of age, the ultrasound images could not be recovered due to excessive dorsal fat layer (>20 mm). Therefore, the total of animals scanned near slaughtering with valid figures was 9 purebred Wagyu, 19 Wangus, and 29 ACL steers (Table 3).

Ultrasound scanning traits related to growth and fat deposition [41] were:

- Rump fat thickness (RF, mm), at the “P8” rump site. Measured at the level of the rump, at the intersection of the *gluteus medius* and *biceps femoris* muscles.
- Depth of the *gluteus medius* muscle (GMD, in mm). Measured at point P8.
- Ribeye area (REA, in cm2) of the Longissimus thoracis muscle measured between the 12–13th ribs.
- Back fat (BF, mm). It was 12–13th rib fat thickness.
- Percentage of intramuscular fat estimation (marbling; IMF) of the *Longissimus lumborum* muscle was measured between the 12–13th ribs as a numerical value given by the ultrasound equipment software and positively correlated to intramuscular fat.

### 2.3. Metabolic Status of the Animals

Before slaughtering, blood samples were extracted for sanitary reasons by the official veterinarians, and the authors received an aliquot for metabolic assessment. Once obtained, plasma was separated by centrifugation at 4500× *g* for 15 min and stored in polypropylene vials at −80 °C until later analysis. Parameters of plasma total cholesterol (TC, mg/dL), triglycerides (TG, mg/dL), high- (HDL, mg/dL) and low-density lipoprotein cholesterol (LDL, mg/dL), glucose (GLU, mg/dL), fructosamine (FRU, mg/dL), lactate (LAC, mg/dL), β-hydroxy butyrate (BHB, mmol/L), non-esterified-fatty acid (NEFA mmol/L) and urea (UR, mg/dL) were assessed with clinical chemistry analyzer (Konelab 20; Thermo Fisher Scientific, Waltham, MA, USA), according to the manufacturer’s instructions. Plasma leptin concentration (LEP, µg/mL) was determined with a multispecies ELISA kit (Demeditec Diagnostics GmbH, Kiel, Germany; assay sensitivity 0.25 µg/mL and intra-assay variation coefficient < 15%).

### 2.4. Meat Nutritional Analyses

Meat analyses were performed immediately after reception at the laboratory Labocor Analitica on fresh samples. Samples were minced and homogenized. Crude protein content was determined by Kjeldahl [42] with a conversion factor of 6.25, and total fat was determined by ethyl ether extraction with previously acid-hydrolyzed samples (Soxhlet technique [43]). Moisture was gravimetrically measured by drying at 103 ± 2 °C [44], and ash was gravimetrically assessed after combustion at 550 °C in a muffle furnace [45] to a constant weight. Energy content (kcal) was calculated based on 100 g portion using according to the equation of Merrill and Watt [46] as the following: energy value (kcal/100 g) = (Protein% × 4) + (Fat% × 9) + (Carbohydrate% × 4). Cholesterol in meat was assessed with the enzymatic method (Kit Boehringer Mannheim) and read by spectrophotometry. pH- were quantified with a glass electrode (FC2323) specifically designed for meat, introduced 1 cm into the meat, performing 3 measurements/piece, and recalibrated by each sample.

Fatty acid content of beef was determined by extracting the fat fraction, following the method of ISO [47] for determining FA methyl esters by gas chromatography, set up, and verified. Only cis-isomers of fatty acids were detected. Fatty acids were expressed as a concentration in fresh meat and/or as a percentage of the total amount of the identified fatty acids. The total contents of saturated fatty acids (SFAs), unsaturated fatty acids (UFAs), monounsaturated fatty acids (MUFAs), polyunsaturated fatty acids (PUFAs), and ω–3 and ω–6 PUFAs were calculated.

In addition, the health-related indexes of the lipid fraction were assessed by the following calculations:

- ω–6/ω–3 ratio: ω–6 to ω–3 fatty acids ratio (optimum value ≈< 4; although it is a discussed cut-off) [48].
  - Σω–6 PUFA/Σω–3 PUFA
- Atherogenic index (AI [49]; the lower the healthier).
  - AI = [12:0 + (4×14:0) + 16:0]/(Σω–3 PUFA + Σω–6 PUFA + ΣMUFA)
- Thrombogenicity index (TI [49]; the lower the healthier).
  - TI = (14:0 + 16:0 + 18:0)/[(0.5 × ΣMUFA) + (0.5 × Σω–6 PUFA) +(3 × Σω–3 PUFA) + (Σω–3 PUFA/Σω–6 PUFA)]
- Hypo-/hypercholesterolemic ratio (h/H; modified from Fernández et al. [50]; the higher the healthier).
  - h/H = [(C18:1 + C18:2n–6 + C18:3n–6 + C18:3n–3 + C20:3n–6 + C20:4n–6 + C20:5n–3 + C22:4n–6 + C22:5n–3 + C22:6n–3)/(C14:0 + C16:0)].

### 2.5. Amino Acids Assessment

A representative subset of the entrecote samples of each breed group was randomly selected (n = 13/24 WY, 12/29 WN and 8/29 ACL). Samples were hydrolyzed with HCL 6N, at the Laboratory of the Veterinary Faculty at the Universidad Católica of Valencia (Valencia, Spain), by high-performance liquid chromatography (HPLC), with the derivatization method (Waters AccQ-Tag reagent kit). Then, the amino acids were separated on a reverse phase C18 column (AccQ Tag, 3.9 × 150 mm, Waters Co, Milford, CT, USA) using a gradient of two eluents from the kit as mobile phase. Three µL of sample were injected for each analysis carried out in 50 min.

The detection of the amino acids was carried out with a fluorescence detector set up to work at 250 and 395 nm (excitation and emission wavelengths, respectively). Amino acids were identified by retention times and quantified by the external standard technique using an amino acid standard [α–aminobutyric acid (αAba)] and processed with the EZChromeElite program (Agilent, Santa Clara, CA, USA). Results were expressed in g/100 g of dry matter.

Tryptophan content was determined following the protocol described by Yust et al. [51], in which alkaline hydrolysis of samples of meat and ashes was performed. In brief, tryptophan was extracted from other amino acids and assessed by reverse phase HPLC and its detection by absorbance set up with 280 nm wavelength. The same reverse phase C18 column was used for separating derivatized amino acids according to the Waters AccQ–Tag method described above. Tryptophan was quantified using a standard curve with different concentrations of tryptophan standard. Results were expressed in g/100 g of dry matter.

### 2.6. Statistical Analyses

Data were analyzed using SPSS^®^ v.25 (IBM, Armonk, NY, USA). Normality of variables was assessed with Kolmogorov–Smirnov and Shapiro–Wilk tests. Most of the variables showed a skewed distribution, and all the variables were reported as median and range instead of average and standard deviation as best measures of centrality and variability for skewed distributions; therefore, non-parametric tests were used for assessing intergroup differences such as Kruskal–Wallis test for independent samples. The comparisons were made among experimental groups (three groups, one full breed: WY and two crossbreeds: WN and ACL), separated by beef cut (sirloin or entrecote) or between cuts (sirloin or entrecote) separated by experimental groups. Potential pairwise relationships among numerical variables of steers characteristics prior slaughtering, blood metabolites, meat nutritional parameters, and amino acid values were assessed with Spearman tests. Differences associated with *p*-values ≤ 0.05 were considered significant.

## 3. Results

### 3.1. Pre-Slaughter Metabolic Data

WY and WN steers showed levels of metabolites in plasma that were not statistically different (Table 4). Concentrations of all lipid-related metabolites, except NEFA and LDL, were higher in WY and WN steers than in ACL animals. Conversely, glucose values were lower in WY and WN steers. Levels of the hormone leptin, which are related to the lipid profile, were higher in WN than in ACL steers.

### 3.2. Nutritional, Fatty Acid, and Amino Acid Profiles in Beef Samples

Nutritional analyses showed that for both meat cuts, WY and WN steers showed higher fat infiltration than ACL animals, including 2 to 2.5-fold higher IMF (in the fresh matter; Table 5). However, ACL meat had a higher content of protein and lower content of saturated fatty acids in both cuts of meat. Entrecote samples showed more IMF and less SFA in all breeds (Table 5).

The relative content of saturated fatty acids, expressed as the percentage of the total amount of identified fatty acids, varied depending on the cut of meat. Only in ACL animals, the amount of PUFA and the ratio ω–6/ω–3 were significantly higher in sirloin than in entrecote (Table 6). Entrecote samples from ACL steers contained more saturated fatty acids than the corresponding cut from the other two breeds, with the exception of stearic acid (C18:0, Table 7). However, this pattern was absent in sirloin samples, where only stearic acid values differed, with WN steers presenting the lowest level.

The relative content of unsaturated fatty acids was higher in sirloin from WN steers and lower in entrecote from ACL steers (Table 6), with WY and WN samples showing the highest concentrations of oleic acid (C18:1; Table 7) and linoleic acid (C18:2n–6). Conversely, the content of linoleic acid (C18:2n–6) was highest in ACL steers. C17:1 was the most abundant fatty acid in sirloin from WN animals.

Next, we correlated the fat characteristics of meat with healthiness indexes for humans. The ratio of monounsaturated to saturated fatty acids (MUFA/SFA) was higher in WY and WN than in ACL meat (Table 6). The ACL entrecote showed the lowest hypocholesterolemic/hypercholesterolemic index, indicating less healthy meat, while sirloin did not differ substantially in this index across the breeds. Moreover, for both cuts of meat, ACL steers showed the highest thrombogenicity index, indicating less healthy meat, and their entrecote showed the highest atherogenic index. Consistently, ACL meat showed the highest ratio of ω–6 to ω–3 than Wagyu in both cuts, and Wangus in sirloin, which ideally should be <4.

The amino acid content of entrecote samples did not differ among breed/crossbreed (Table 8), except that ACL steers showed higher absolute levels of arginine and threonine than the other two breeds and higher leucine content than Wagyu due to the higher crude protein content.

### 3.3. Relationships between Variables

**Figure 1:**
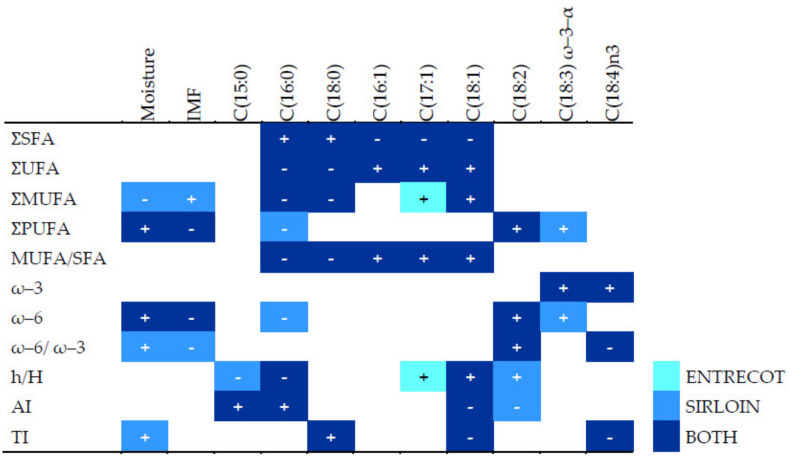
Spearman coefficients for statistically significant pairwise correlations (*p* < 0.001, with absolute value > 0.4) between human health-related indexes, total content of fatty acid types, and specific fatty acids, analyzed separately by meat cut, entrecote and sirloin samples, regardless of experimental group. The “+” symbol represents a positive correlation and “-”, a negative correlation. AI: atherogenic index; h/H: hypocholesterolemic/hypercholesterolemic index; IMF: intramuscular fat in fresh matter; MUFA: monounsaturated fatty acid; PUFA: polyunsaturated fatty acid; SFA: saturated fatty acid; TI: thrombogenicity index; UFA: unsaturated fatty acid; Ʃ: total sum of; ω–3: omega-3 fatty acids; ω–6: omega-6 fatty acids; ω–6/ω–3: ratio of ω–6 to ω–3 fatty acids.

In sirloin, the IMF content samples correlated positively with the total content of monounsaturated fatty acids due to the oleic acid content and inversely with the content of polyunsaturated fatty acids or PUFAs (Figure 1). In entrecote and sirloin, the human health-related indexes AI and TI correlated negatively with unsaturated fatty acids but positively with saturated fatty acids, while the opposite was true for the h/H ratio.

Among blood metabolites, IMF correlated positively with total cholesterol and high-density lipoprotein cholesterol, regardless of the meat cut (Table 9). β-hydroxybutyrate correlated with moisture (negatively) and IMF (positively), but only in sirloin, while the negative correlation between the total content of PUFAs and urea was found only in entrecote. Plasma total cholesterol and HDL were revealed to be positively associated with the IMF in beef in both cuts and negatively with the amount of ω–6 PUFAs in entrecote and sirloin, independently of breed/crossbreed.

**Figure 2:**
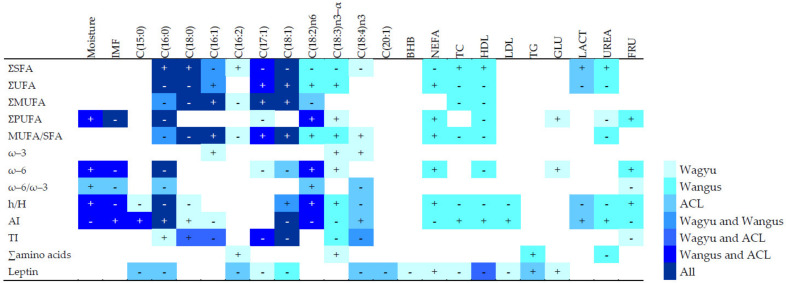
Pairwise correlations among health-related indexes, total content of different fatty acid types, specific fatty acids, and blood metabolites analyzed separately by experimental group, in Wagyu, Wangus, and Angus-by-Charolais or Limousine steers, regardless of meat cut. Only correlations for which Spearman’s coefficient had an absolute value > 0.4 and *p* < 0.001 are shown. “+” symbol indicates a positive correlation; “-” indicates a negative correlation. AI: atherogenic index; BHB: β-hydroxybutyrate; FRU: fructosamine; GLU: glucose; HDL: high-density lipoprotein cholesterol; h/H: hypocholesterolemic/hypercholesterolemic index; IMF: intramuscular fat in fresh matter; LACT: lactate; LDL: low-density lipoprotein cholesterol; MUFA: monounsaturated fatty acid; NEFA: non-esterified fatty acid; PUFA: polyunsaturated fatty acid; SFA: saturated fatty acid; TC: serum total cholesterol; TG: triglycerides; TI: thrombogenicity index; UFA: unsaturated fatty acid; ∑: total content; ω–3: omega-3 fatty acids; ω–6: omega-6 fatty acids; ω–6/ω–3: ratio of ω–6 to ω–3 fatty acid.

Figure 2 summarizes the significant pairwise correlations between variables by breed/crossbreed. In WY meat, moisture and IMF did not correlate with health-related indexes. In WN or ACL meat, increased moisture and decreased IMF correlated with better health-related indexes. In meat from WN and ACL steers, but not WY animals, moisture and IFM correlated negatively with the atherogenic index and positively with the ratio h/H.

Content of specific fatty acids correlated with the corresponding total amounts. In all breeds, higher content of palmitic acid (C16:0), the most frequent saturated fatty acid, was associated with worse health-related indexes. The content of other fatty acids correlated differently with health-related indexes depending on the experimental group. In WY and ACL meat, the content of stearic fatty acid (C18:0) was positively correlated with the TI index. In WY meat, the content of linoleic acid [C18:2n–6] and linolenic acid [C18:3n–3] did not correlate with h/H, TI, or AI. However, a robust positive correlation with health-related indexes was observed in WN and ACL steers for the linoleic acid, while such a relationship was observed for linolenic acid, but only in WN meat. The stearidonic fatty acid [C18:4n–3] correlated negatively with TI in WY and WN steers, while it was positively related to h/H and AI in ACL steers.

More pre-slaughter metabolic parameters correlated with fatty acid composition and leptin content in meat from WN animals than in meat from WY or ACL steers (UREA, TC, and HDL positively correlated to saturated fatty acids). Leptin levels, although highest in WN animals, significantly correlated with NEFA and glucose metabolic parameters in purebred WY steers, while leptin correlated positively in ACL animals only with triglycerides (Figure 2).

## 4. Discussion

Wagyu and Wangus steers are of high commercial interest worldwide because of their high beef quality, due in part to high IMF content. The impact of different fattening systems on beef quality and fat profile is poorly understood. Therefore, we aimed to describe the pre-slaughter metabolic parameters, beef quality, and fat profile of the final marketable meat samples from three high-marbling bovine breeds/crossbreeds raised under the same Spanish fattening system but slaughtered at different ages. We also analyzed pairwise relationships among variables. As we hypothesized, pure and crossbred WY steers presented higher IMF content, lower crude protein content, and a healthier fatty acid profile than the European ACL crossbred steers.

Regarding the pre-slaughter metabolic biomarkers, ACL animals showed lower levels of blood plasma metabolites related to lipid metabolism, including leptin, than WY and WN animals. The exception was LDL. These differences may be due to the experimental group but also to the different slaughter ages (ACL animals were slaughtered with an age of at least 10 months younger than WY and WN steers), a factor demonstrated to alter the metabolic status of cattle [28]. The only metabolic parameter that was higher in ACL animals than in the other was plasma glucose concentration, which supports previous findings demonstrating higher insulin levels and lower plasma glucose in WY steers than in European crossbreeds, and a positive correlation between IMF and insulin in cattle [30,52]. We found that pre-slaughtering blood plasma BHB correlated negatively with beef moisture and positively with IMF, independently of breed/crossbreed. The production of BHB inhibits some class I histone deacetylases [53], reducing lipolysis in adipocytes. The availability of fatty acids in the liver, which is strongly regulated by insulin, is a critical determinant of ketogenesis, so the reduction of lipolysis may act as a feedback mechanism to limit BHB production [54]. Such feedback may explain the positive correlation between BHB and IMF: increased BHB reduces lipolysis and therefore increases IMF. It may also explain the negative correlation between BHB and moisture: moisture correlates inversely with IMF. Pre-slaughtering values of HDL related to lipid beef characteristics, underscoring a possible metabolic biomarker directly related to beef quality, i.e., foreseeing a high level of IMF and ω–6 PUFA in beef, result highly interesting. Moreover, the correlations between metabolites and fat profiles differed clearly among experimental groups. In general, WN meat showed stronger correlations between metabolic parameters and fatty acids than WY meat (Figure 2). This may indicate that the metabolism of WY animals is better adapted to high body fat than the metabolism of WN animals.

As expected, WY and WN steers showed more fat infiltration in both meat cuts due to their typical Wagyu beef characteristics, and accordingly, the energy content of Wagyu and Wangu beef was significantly higher than that of ACL steers, and the absolute amount of SFA per 100 g of meat was the lowest in the ACL beef samples. We did not observe differences in IMF between purebred WY and crossbred WN animals. Previous work [55] confirmed that crossbreds with WY show stronger IMF content and greater tenderness and marbling score [56], which would explain the high IMF values in our WN steers. Therefore, our work illustrates that the relationships among the meat characteristics that we examined depend on the experimental group (i.e., on breed/crossbreed), even when diet and management are the same. These differences could also relate to the different slaughtering age (linked, in turn, to the group) effects that we cannot separate in this study.

Regarding the assessment of the lipid fraction in beef, among the three experimental groups in our study, ACL steers showed the lowest levels of MUFAs. Previous work found higher MUFA values in subcutaneous fat from WY steers (60.7%) and crossbred WN steers (60.1%) than from Holstein animals (57.6%) [57]. This result reflects the higher ratio of C18:1 to C18:0 desaturases in the WY breed, which leads to higher oleic acid content [58]. Interestingly, our WN crossbred showed even higher MUFA values than WY in sirloin; the WY values were similar to those in a previous study [57]. On the other hand, other authors did not observe such differences when they compared WY animals with the genetically similar Hanwoo and Jeju Black breeds [59]. In our work, this specific high-olein diet may have promoted a higher content of MUFAs in WY and WN steers. Similar results have been reported in steers fed with diets rich in oleic acid, which increased the oleic acid content in meat fat from 1.72 to 4.22% [60]. This may have important implications for the marketability of this meat since consumption of Hanwoo beef from animals with high oleic acid content might reduce the risk of cardiovascular disease [61]. The link between a grain-fed diet and higher, healthier levels of MUFAs in beef was also observed previously in various breeds, such as Angus, Hereford, and Hanwoo, among others [62].

Angus-by-Charolais or Limousine beef in our study showed the highest content in PUFAs and the highest ω–6/ω–3 ratio across all three breed/crossbreds, with nearly doubling the content of linoleic acid (C18:2n-6). In contrast, the entrecote pieces of ACL showed the highest content of saturated fatty acids, similar to that reported in former reports [63,64]. In the current study, a high-olein diet appeared to induce high levels of PUFAs in all three breeds/crossbreds. These values were like those previously described in beef cattle of other breeds (4–5%) [65,66] and higher than those reported in grain-fed WY steers (2.6–3.5%) [56,59]. It seems, therefore, that a high-olein diet improves beef quality more intensively, leading to levels of PUFAs and ω–6/ω–3 ratio healthier for humans in WY steers than in European crossbred animals.

The health-related indexes AI and TI in our work correlated negatively with the content of unsaturated fatty acids (improving the effect for healthiness in humans) and positively with the content of saturated fatty acids (worsening the effect for healthiness in humans). These relationships seem logical given that the indexes are calculated in a way that saturated fatty acid content is penalized due to its assumed adverse effects on human health. One study has linked worse AI and TI indexes to higher saturated fatty acid content in beef as well as a greater risk of cardiovascular disease in humans who consume that beef [10]. However, the inclusion of beef in a healthy, balanced diet does not necessarily increase the risk of cardiovascular disease [13], especially if the fatty acids in the meat are predominantly unsaturated.

Although the two cuts of meat in our study are similarly highly regarded on the market, we found interesting differences in nutritional parameters and in the lipid fraction between cuts from the same breed/crossbreed, some of them previously found [67], as well as in correlations between those variables and health-related indexes. Independently of the experimental group, entrecote showed more IMF than sirloin pieces and, accordingly, more energy content and SFA in g/100 g of meat. In the lipid fraction, the main difference between sirloin and entrecote differed by group, with ACL animals showing higher PUFA and lower SFA contents in sirloin than in entrecote. The relationship among fatty acids also varied by meat cut. The higher content of total fat and total saturated fatty acids in entrecote probably weakened some relative relationships among specific MUFAs, PUFAS, and health-related indexes, indeed observed in sirloin. For example, content in ω–6 fatty acids correlated negatively with palmitic acid and positively with linolenic acid in sirloin but not in entrecote. Only in entrecote did we find correlations of lower values of AI with higher values of linoleic acid and higher values of h/H, indicating healthier beef, with linoleic acid being a potential marker. In fact, linoleic acid is known to be beneficial to human health [68], and it is valued by beef consumers [69]. Therefore, the cut should always be taken into account when analyzing the beef quality and healthiness of humans.

Congruently with a lower absolute content in intramuscular fat, Angus-by-Charolais or Limousine beef samples had more crude protein per 100 g of entrecote and a higher absolute content of several amino acids. Unfortunately, none of the three types of beef showed higher content of essential amino acids. Moreover, a higher protein level may not always be desirable: for example, higher levels of leucine and methionine in ACL beef can lead to a bitter taste in consumers [57]. Wagyu and Wangus beef may be superior to ACL beef in this regard. In addition, WY and WN beef showed a higher ratio of MUFAs to saturated fatty acids, indicating better organoleptic qualities [70]. The three kinds of beef should be compared in taster panel studies.

Finally, the health-promoting value of meat can be assessed based on indexes of its various long-chain fatty acids [71]. As we hypothesized, the different groups in our study showed different indexes linked to different intramuscular lipid fractions. Congruently with the higher content in PUFAs and higher ω–6/ω–3 ratio in beef, ACL entrecote showed the lowest h/H index and the highest atherogenic index, and both meat cuts from ACL steers showed the highest thrombogenicity index. These results support the idea that beef quality strongly depends on the breed/crossbreed [72,73]. In fact, specific genes in WY steers have been positively linked to high fat deposition [74], and the maternal gametic effect may also contribute to fat deposition [75]. This study implies that the fat composition of beef influences many aspects of its quality and health value [4,5,6]. In a previous study, adding olive oil to the diet of Blonde d’Aquitaine steers led to a similar atherogenic index as that in our ACL bulls (0.6), which further improved when soy oil was used instead of olive oil [27]. In the current study, a high-olein diet led to better entrecote fat in WY steers than in other groups, as well as lower AI and TI (Table 6). In general, lower atherogenic and thrombogenicity indexes, a higher h/H ratio, and a ω–6/ω–3 ratio lower than four are desirable [48]. This kind of fat profile lowers LDL [19], increases HDL, and decreases triglycerides in humans [20], although the magnitude of the health benefit requires further study [76]. Moreover, based on the lipid fraction, beef fat from WY may be healthier for humans than beef fat from European crossbreeds raised under the same conditions. These considerations also suggest that raising WY steers on pasture or feeding them forage may further improve the health-related indexes of WY beef, as suggested before [77], which is an interesting question for further research [78].

The information about the influence of breed/crossbreed and management systems on the lipid fraction of meat is further scarce, and although recent efforts in “foodomics” have brought some light to this topic [79], further research is required.

## 5. Conclusions

Differences among experimental groups in certain metabolic parameters were found, probably linked not only to the breed or crossbreed but also to slaughtering age. Pre-slaughtering values of plasma HDL underscored as a possible metabolic biomarker directly related to beef quality and concretely to intramuscular fat. Although the fat profile and nutritional content of beef depend on the cut, on the breed/crossbreed, and on the slaughtering age, purebred WY and their crossbred WN, raised in Spain under fattening conditions characterized by olein-rich diets, no exercise restriction, and high animal welfare, achieve high IMF content and quality that are similar to those of animals fattened in Japan.

## Figures and Tables

**Table 1:** Diet composition (percentage of dry matter) given to all steers in the study during the final phase of the fattening period.

Nutrient	Finishing Dry-TMR
Humidity (%)	11.35
Dry matter (%)	88.65
Crude fiber (%)	11.15
Ash (%)	5.58
NDF (%)	26.43
Crude protein (%)	13.19
Crude fat (%)	6.98
ADF (%)	14.16
Ca (%)	0.628
P (%)	0.292
Na (%)	0.219
Cl (%)	0.419
Mg (%)	0.219
K (%)	0.778
S (%)	0.172
Vitamin E (mg/kg)	41.62
Vitamin A (KUI/kg)	4.23
Vitamin D (KUI/kg)	1.18
NFC	47.83
Starch (%)	40.46
ME (kcal/kg)	3026.4
TDN (%)	80.93

ADF: acid detergent fiber; Ca: calcium; Cl: chlorine; K: potassium; ME: metabolizable energy; Mg: magnesium; Na: sodium; NDF: neutral detergent fiber; P: phosphorus; S: sulfur; NFC: non-fibrous carbohydrates, calculated according to Institut National de la Recherche Agronomique (INRA) guidelines; TDN: total digestive nutrients; TMR: total mixed ration.

**Table 2:** Fatty acid profile (expressed in percentage of total fatty acids) of the diet used to feed all steers in the study during the final phase of the fattening period.

Fatty Acid	Finishing Dry-TMR
Saturated fatty acids (SFA, %)	
C14:0	0.19
C16:0	15.71
C17:0	0.16
C18:0	5.16
C20:0	0.55
Unsaturated fatty acids (UFA, %)	
C16:1	0.76
C17:1	0.11
C18:1	41.83
C18:2n-6	32.55
C18:3n-3	2.69
C20:1	0.29
Fatty acid profile	
ƩSFA (%)	21.77
ƩMUFA (%)	42.99
ƩPUFA (%)	35.24
MUFA/SFA	1.97
ω–3 (%)	2.69
ω–6 (%)	32.55
ω–6/ω–3 (%)	12.10

MUFA: monounsaturated fatty acids; PUFA: polyunsaturated fatty acids; SFA: saturated fatty acids; TMR: total mixed ration; UFA: unsaturated fatty acids; Ʃ; total sum of; ω–3: omega-3 fatty acids; ω–6: omega-6 fatty acids; ω–6/ω–3 ratio: ω–6 to ω–3 fatty acids ratio.

**Table 3:** Characteristics of steers measured prior slaughtering, including last in vivo ultrasound evaluation of muscle and fat deposition (median and interquartile range).

Characteristic	WY		WN		ACL		*p*-Value
	n	Median (IQR)	n	Median (IQR)	n	Median (IQR)	
Age (mo.)	24	38.4 (34.9–40.3) ^a^	29	30.6 (26.9–36.5) ^b^	29	20.3 (19.0–22.7) ^c^	**<0.001**
Weight (kg)	15	840 (785–895)	14	832 (802–875)	29	780 (715–852)	0.070
Height (cm)	15	147.0 (145.0–150.0)	14	146.0 (141.5–147.5)	29	143.0 (140.0–146.0)	0.100
BF (mm)	9	18.6 (16.9–23.5)	16	22.5 (19.3–23.6)	29	19.8 (15.3–24.8)	0.500
REA (cm^2^)	9	91.7 (84.9–104.8) ^a^	16	100.0 (88.8–111.4) ^a^	29	110.3 (101.7–118.9) ^b^	**0.005**
RF (mm)	9	14.1 (13.1–20.1)	16	14.1 (9.9–19.0)	29	15.9 (13.4–18.1)	0.600
GMD (mm)	9	92.0 (87.1–98.4) ^a^	16	101.4 (95.4–107.6) ^a^	29	116.4 (107.5–123.4) ^b^	**<0.001**
IMF	9	7.9 (7.2–8.1) ^a^	16	6.8 (6.4–7.8) ^b^	29	6.6 (6.1–7.1)^b^	**0.006**

ACL: Angus-by-Charolais or Limousine animals; BF: back fat thickness measured between the 12–13th ribs; GMD: depth of the *gluteus medius* muscle measured at point “P8” on the rump; IMF: in vivo intramuscular fat estimation (numerical values provided by the software); IQR: interquartile range; REA: ribeye area of the *Longissimus thoracis* measured between the 12–13th ribs; RF: rump fat thickness at point “P8”; WY: Wagyu; WN: Wagyu-by-Angus (Wangus). Different superscripts in values of the same row indicate significant differences with the *p*-value of the last column.

**Table 4:** Plasma metabolites determined one week prior to slaughter in steers of different breed/crossbreed (median and interquartile range).

Metabolite	WY	WN	ACL	*p*-Value
TC (mg/dL)	163.3 (150.0–194.0) ^a^	145.5 (124.5–162.7) ^ab^	111.5 (98.0–137.0) ^b^	**<0.001**
HDL (mg/dL)	71.0 (61.1–90.0) ^a^	54.2 (47.2–80.5) ^a^	42.5 (39.0–53.5) ^b^	**<0.001**
LDL (mg/dL)	17.3 (12.2–20.0)	19.8 (17.0–22.3)	17.5 (15.3–24.2)	0.300
TG (mg/dL)	29.0 (26.1–37.2) ^a^	28.3 (25.5–43.0) ^a^	19.1 (16.4–35.2) ^b^	**0.040**
BHB (mmol/L)	0.45 (0.38–0.58) ^a^	0.43 (0.35–0.53) ^a^	0.35 (0.27–0.41) ^b^	**<0.001**
NEFA (mmol/L)	0.16 (0.11–0.39)	0.21 (0.15–0.38)	0.26 (0.2–0.3)	0.500
GLU (mg/dL)	76.3 (73.1–91.2) ^a^	81.8 (76.8–91.5) ^a^	92.4 (85.0–97.4) ^b^	**0.010**
FRU (mg/dL)	294.0 (274.7–310.7)	289.0 (270.5–315.5)	300 (278.5–329.0)	0.400
LAC (mg/dL)	20.2 (16.0–39.5)	19.6 (12.8–35.3)	20.0 (13.2–32.1)	0.700
UR (mg/L)	27.2 (23.0–34.2) ^ab^	29.5 (23–42.1) ^a^	23.5 (20.3–28.1) ^b^	**0.040**
Leptin (µg/mL)	12.1 (0.36–14.9) ^ab^	17.9 (11.0–26.2) ^a^	8.9 (5.5–11.7) ^b^	**0.022**

ACL: Angus-by-Charolais or Limousine animals; BHB: β–hydroxybutyrate; FRU: fructosamine; GLU: glucose; HDL: high-density lipoprotein cholesterol; LAC: lactate; LDL: low-density lipoprotein cholesterol; NEFA: non-esterified fatty acid; TC: total cholesterol; TG: triglycerides; UR: urea; WN: Wangus; WY: Wagyu. Values are expressed as median (interquartile range). Different superscripts in values of the same row indicate significant differences with the *p*-value in the last column.

**Table 5:** Nutritional composition of fresh sirloin and entrecote samples from Wagyu (WY), Wangus (WN), and Angus-by-Charolais or Limousine (ACL) steers (median and interquartile range).

	Sirloin				Entrecote			
Parameter	WY	WN	ACL	*p*-Value	WY	WN	ACL	*p*-Value
pH	5.4 (5.3–5.56) ^a^	5.6 (5.5–5.8) ^b^	5.7 (5.4–5.8) ^b^	**0.014**	5.5 (5.4–5.6) ^a^	5.6 (5.5–5.8) ^b^	5.7 (5.6–5.8) ^b^	**0.003**
Moisture (%)	59.4 (56.5–62.6) ^a^	61.2 (56.9–66.6) ^a^	72.2 (70.1–74.2) ^b^	**<0.001**	54.9 (50.4–60.0) ^a^	59.5 (55.4–63.5) ^a^	69.1 (67.3–71.3) ^b^	**<0.001**
IMF (%)	20.5 (19.0–26.1) ^a^	18.0 (11.6–24.7) ^a^	6.1 (3.7–8.3) ^b^	**<0.001**	26.9 (21.1–32.6) ^a^	20.9 (16–26.6) ^a^	8.6 (5.6–11.6) ^b^	**<0.001**
P (g/100 g)	18.4 (17.1–19.4) ^a^	18.6 (18.1–20.5) ^a^	20.5 (19.6–21.4) ^b^	**<0.001**	16.7 (15.5–18.3) ^a^	18.2 (17.2–20) ^b^	21.05 (20.5–21.8) ^a^	**<0.001**
Ash (%)	0.9 (0.8–1.0) ^a^	1.0 (0.9–1.1) ^a^	1.2 (1.0–1.3) ^b^	**<0.001**	0.8 (0.7–1.0) ^a^	0.9 (0.8–1.0) ^a^	1.1 (1.0–1.2) ^b^	**<0.001**
E (kcal/100 g)	259 (227–309) ^a^	241 (185–284) ^a^	137 (113–157) ^b^	**<0.001**	312 (262–361) ^a^	269 (228–308) ^a^	162 (141–190) ^b^	**<0.001**
E (kJ/100 g)	1074 (944–1282) ^a^	1002 (772–1175) ^a^	574 (478–656) ^b^	**<0.001**	1291 (1085–1494) ^a^	1115 (947–1275) ^a^	681 (593–791) ^b^	**<0.001**
SFA (g/100 g)	9.3 (6.9–11.4) ^a^	7.3 (4.4–10.3) ^a^	2.3 (1.6–3.8) ^b^	**<0.001**	11.5 (8.3–13.9) ^a^	8.7 (6.8–11.4) ^a^	3.9 (2.5–5.3) ^b^	**<0.001**
Cholesterol (%)	0.65 (0.44–1.09) ^ab^	0.52 (0.37–0.64) ^a^	0.66 (0.51–0.87) ^b^	**0.030**	0.81 (0.59–1.02)	0.70 (0.49–0.94)	0.69 (0.55–1.10)	0.500

E: energy content in 100 g of meat; IMF: intramuscular fat in fresh matter (%); P: crude protein content (g/100 g); SFA: saturated fatty acids (g/100 g). Values are expressed as median (interquartile range). Different superscripts in values of the same row indicate significant differences with the *p*–value of the corresponding column.

**Table 6:** Fatty acid profile (percentage of total fatty acids) and health-related indexes of sirloin and entrecote meat from Wagyu (WY), Wangus (WN), and Angus-by-Charolais or Limousine (ACL) steers (median and interquartile range).

Parameter *	Sirloin				Entrecote			
	WY	WN	ACL	*p*-Value	WY	WN	ACL	*p*-Value
ƩSFA (%)	44.4 (40.9–46.2) ^a^	42.1 (37.2–44.) ^b^	43.8 (41.8–47.2) ^a^	**0.02**	43.2 (40.6–44.9) ^a^	41.67 (36.5–43.0) ^a^	45.6 (43.0–47.5) ^b^	**<0.001**
ƩMUFA (%)	50.3 (48.7–53.3) ^a^	52.5 (50.1–54.9) ^a^	46.0 (44.8–48.1) ^b^	**<0.001**	52.1 (49.0–55.3) ^a^	53.1 (50.8–54.9) ^a^	48.36 (45.5–50.0) ^b^	**<0.001**
ƩPUFA (%)	4.7 (4.1–6.2) ^a^	4.4 (3.8–6.6) ^a^	7.11 (6.2–10.1) ^b^	**0.001**	4.13 (3.5–5.2) ^a^	4.3 (3.8–5.1) ^a^	5.76 (5.0–6.3) ^b^	**<0.001**
MUFA/SFA	1.2 (1.1–1.3) ^a^	1.3 (1.1–1.5) ^b^	1.1 (1.0–1.1) ^c^	**<0.001**	1.2 (1.1–1.4) ^a^	1.3 (1.2–1.5) ^a^	1.1 (1.0–1.2) ^b^	**<0.001**
ω–3 (%)	0.67 (0.55–0.78)	0.59 (0.43–0.68)	0.58 (0.49–0.77)	0.410	0.66 (0.49–0.77)	0.55 (0.48–0.63)	0.56 (0.45–0.66)	0.200
ω–6 (%)	3.6 (3.1–5.1) ^a^	3.5 (3.1–5.5) ^a^	6.1 (5.4–9.4) ^b^	**<0.001**	3.2 (2.6–4.3) ^a^	3.4 (3.1–4.2) ^a^	4.8 (4.3–5.5) ^b^	**<0.001**
ω–6/ω–3	6.5 (4.7–9.3) ^a^	8.1 (5.5–9.6) ^a^	11.5 (8.2–16.8) ^b^	**0.002**	5.1 (4.1–7.1) ^a^	7.0 (5.5–9.5) ^ab^	8.9 (6.3–10.6) ^b^	**0.004**
AI	0.60 (0.54–0.65)	0.54 (0.47–0.66)	0.61 (0.53–0.68)	0.180	0.60 (0.51–0.65) ^a^	0.55 (0.47–0.63) ^a^	0.69 (0.61–0.75) ^b^	**<0.001**
TI	0.88 (0.77–1.14) ^a^	0.92 (0.79–0.99) ^a^	1.1 (1.0–1.2) ^b^	**<0.001**	0.82 (0.71–1.0) ^a^	0.92 (0.82–1.0) ^a^	1.1 (1.0–1.2) ^b^	**<0.001**
h/H	1.9 (1.8–2.1)	2.1 (1.8–2.4)	1.9 (1.7–2.1)	0.140	1.9 (1.8–2.2) ^a^	2.1 (1.8–2.3) ^a^	1.7 (1.6–1.9) ^b^	**<0.001**

* Only cis isomers of the FA were detected. AI: atherogenic index; h/H: hypocholesterolemic/hypercholesterolemic index; IMF: intramuscular fat; MUFA: monounsaturated fatty acid; PUFA: polyunsaturated fatty acid; SFA: saturated fatty acid; TI: thrombogenicity index; UFA: unsaturated fatty acid; Ʃ; total sum of; ω–3: omega-3 fatty acids; ω–6: omega-6 fatty acids; ω–6/ω–3 ratio: ω–6 to ω–3 fatty acids ratio. Values are expressed as median (interquartile range). Fatty acids values are relative percentages of the total amount of identified fatty acids. Different superscripts in values of the same row indicate significant differences with the *p*–value of the last column.

**Table 7:** Percentage of saturated and unsaturated fatty acids in sirloin and entrecote meat from Wagyu (WY), Wangus (WN), and Angus-by-Charolais or Limousine (ACL) steers, expressed as a percentage of the total amount of fatty acids detected (median and interquartile range).

	Sirloin				Entrecote			
Fatty Acid *	WY	WN	ACL	*p*-Value	WY	WN	ACL	*p*-Value
Saturated fatty acids (SFA, %)								
C14:0	2.2 (1.9–2.4)	2.1 (1.7–2.5)	2.1 (1.8–2.5)	0.860	2.2 (2.0–2.4) ^a^	2.1 (1.7–2.3) ^a^	2.4 (2.0–2.9) ^b^	**0.003**
C15:0	0.32 (0.26–0.28)	0.35 (0.28–0.41)	0.33 (0.30–0.41)	0.410	0.28 (0.22–0.33) ^a^	0.31 (0.27–0.37) ^a^	0.36 (0.31–0.44) ^b^	**0.001**
C16:0	24.1 (22.5–25.0)	23.6 (21.2–25.1)	23.8 (22.9–25.4)	0.590	24.1 (22.2–25.0) ^a^	23.0 (21.7–25.0) ^a^	25.7 (24.8–26.7) ^b^	**0.001**
C17:0	0.74 (0.71–1.03)	0.92 (0.81–1.02)	0.94 (0.84–1.08)	0.210	0.74 (0.64–0.89) ^a^	0.78 (0.67–0.93) ^a^	0.91 (0.82–1.06) ^b^	**0.001**
C18:0	16.6 (14.7–18.5) ^a^	14.5 (12.5–15.8) ^b^	16.6 (15.3–17.9) ^a^	**0.002**	15.2 (12.6–16.6)	14.4 (12.3–16.4)	16.0 (12.7–18.8)	0.300
Unsaturated fatty acids (UFA, %)								
C14:1	0.46 (0.39–0.56)	0.56 (0.4–0.66)	0.43 (0.32–0.55)	0.090	0.57 (0.44–0.7)	0.56 (0.50–0.71)	0.61 (0.43–0.72)	0.800
C16:1	2.7 (2.5–3.2)	3.0 (2.6–3.7)	2.9 (2.5–3.4)	0.330	3.48 (2.95–3.69)	3.3 (2.9–3.8)	3.5 (2.8–4.1)	0.900
C16:2	0.32 (0.25–0.47)	0.40 (0.25–0.49)	0.31 (0.22–0.37)	0.300	0.29 (0.26–0.47)	0.27 (0.23–0.36)	0.27 (0.19–0.40)	0.300
C17:1	0.65 (0.55–0.74) ^a^	0.81 (0.68–0.89) ^b^	0.71 (0.65–0.81) ^a^	**0.001**	0.64 (0.54–0.74)	0.82 (0.62–0.90)	0.71 (0.62–0.87)	0.060
C18:1	46.1 (44.8–48.4) ^a^	47.8 (46.1–50.1) ^a^	41.3 (40.6–43.7) ^b^	**<0.001**	47.47 (44.33–50.25) ^a^	48.4 (46.1–50.2) ^a^	43.3 (40.1–45.0) ^b^	**<0.001**
C18:2n–6	4.0 (3.1–5.1) ^a^	3.5 (3.1–5.5) ^a^	6.1 (5.1–8.0) ^b^	**<0.001**	3.17 (2.64–4.32) ^a^	3.4 (3.2–4.3) ^a^	4.8 (4.1–5.5) ^b^	**0.001**
C18:3n–3	0.27 (0.23–0.33)	0.30 (0.23–0.39)	0.33 (0.29–0.38)	0.110	0.27 (0.24–0.31)	0.26 (0.23–0.33)	0.30 (0.24–0.35)	0.300
C18:4n–3	0.37 (0.31–0.45) ^a^	0.30 (0.22–0.37) ^b^	0.26 (0.22–0.31) ^b^	**0.010**	0.39 (0.34–0.44) ^a^	0.28 (0.20–0.34) ^b^	0.29 (0.21–0.36) ^b^	**<0.001**
C20:1	0.25 (0.19–0.33)	0.24 (0.16–0.27)	0.20 (0.16–0.26)	0.250	0.27 (0.19–0.42) ^a^	0.29 (0.16–0.27) ^a^	0.16 (0.14–0.24) ^b^	**0.02**

* Only cis isomers of the FA were detected. Values are expressed as median (interquartile range). Fatty acids values are relative percentages of the total amount of identified fatty acids. Different superscripts in values of the same row indicate significant differences with the *p*–value of the last column.

**Table 8:** Amino acid profiles (g/100 g) in entrecote from Wagyu (WY), Wangus (WN), and Angus-by-Charolais or Limousine (ACL) steers (median and interquartile range).

Amino Acid	WY	WN	ACL	*p*-Value
Alanine	1.0 (0.87–1.4)	1.2 (1.0–1.3)	1.3 (0.96–1.4)	0.720
Arginine	1.2 (0.92–1.5) ^a^	1.2 (1.0–1.3) ^a^	1.7 (1.5–1.9) ^b^	**0.022**
Aspartame	2.4 (2.0–2.8)	2.6 (2.3–3.1)	2.9 (2.4–3.1)	0.370
Glutamate	4.2 (3.3–4.6)	4.2 (6.8–5.1)	4.8 (4.0–5.2)	0.330
Glycine	1.4 (1.1–1.5)	1.3 (1.2–1.5)	1.5 (1.3–2.2)	0.470
Histidine	0.88 (0.75–1.0)	0.89 (0.79–0.96)	1.1 (0.82–1.2)	0.420
Isoleucine ^†^	1.1 (1.0–1.3)	1.3 (1.1–1.5)	1.5 (1.0–1.7)	0.290
Leucine ^†^	1.7 (1.5–1.8) ^a^	1.9 (1.8–2.0) ^ab^	2.1 (1.9–2.2) ^b^	**0.046**
Lysine ^†^	2.2 (1.8–2.5)	2.3 (2.0–2.7)	2.6 (1.7–2.8)	0.580
Methionine ^†^	0.61 (0.52–0.67)	0.68 (0.58–0.72)	0.83 (0.66–0.95)	0.062
Phenylalanine ^†^	1.1 (0.91–1.2)	1.2 (1.1–1.4)	1.3 (1.1–1.4)	0.200
Proline	1.3 (0.96–1.6)	1.2 (1.1–1.4)	1.4 (1.1–1.9)	0.790
Serine	1.1 (0.92–1.3)	1.1 (0.99–1.3)	1.3 (1.1–1.3)	0.450
Threonine ^†^	0.82 (0.75–1.1) ^a^	0.96 (0.70–1.0) ^a^	1.6 (1.1–1.8) ^b^	**0.012**
Tryptophan ^†^	0.19 (0.16–0.20)	0.20 (0.18–0.23)	0.19 (0.15–0.22)	0.410
Tyrosine	0.94 (0.77–1.1)	1.2 (0.97–1.3)	1.3 (0.74–1.4)	0.100
Valine ^†^	1.4 (1.2–1.5)	1.4 (1.3–1.6)	1.6 (1.3–1.8)	0.400
Total essential amino acids	9.0 (7.8–10.1)	10.1 (8.7–11.1)	11.4 (8.9–12.5)	0.210

^†^ Essential amino acids. Values are expressed as median (interquartile range). Different superscripts in values of the same row indicate significant differences with *p*–value in the last column.

**Table 9:** Spearman coefficients for statistically significant pairwise correlations (*p* < 0.001) between human health-related indexes and blood metabolites in entrecote and sirloin samples.

Entrecote	BHB	TC	HDL	Urea
Moisture		−0.481	−0.564	
IMF (fresh)		0.465	0.537	
ƩPUFA			−0.478	−0.404
ω–6		−0.409	−0.486	
**Sirloin**				
Moisture	−0.541	−0.413	−0.504	
IMF (fresh)	0.556	0.412	0.500	
ƩPUFA			−0.485	
ω–6			−0.473	
ω–6/ω–3			−0.453	

Only significant coefficients with absolute values > 0.4 are shown. BHB: β-hydroxybutyrate; HDL: high-density lipoprotein cholesterol; IMF: intramuscular fat in fresh matter; PUFA: polyunsaturated fatty acids. TC: serum total cholesterol; Ʃ: total sum of; ω–6: omega-6 fatty acids; ω–6/ω–3: ratio of ω–6 to ω–3 fatty acids.

## Data Availability

The data presented in this study are available on request from the corresponding author. The data are not publicly available due to farm privacy.
